# Autophagy modulators in type 2 diabetes: A new perspective

**DOI:** 10.1111/1753-0407.70010

**Published:** 2024-12-16

**Authors:** Ayah Talal Zaidalkilani, Hayder M. Al‐kuraishy, Esraa H. Fahad, Ali I. Al‐Gareeb, Yaser Hosny Ali Elewa, Mahmoud Hosny Zahran, Athanasios Alexiou, Marios Papadakis, Ammar AL‐Farga, Gaber El‐Saber Batiha

**Affiliations:** ^1^ Department of Nutrition, Faculty of Pharmacy and Medical Sciences University of Petra Amman Jordan; ^2^ Department of Clinical Pharmacology and Medicine College of Medicine, Al‐Mustansiriyah University Baghdad Iraq; ^3^ Department of Pharmacology and Toxicology College of Pharmacy, Mustansiriyah University Baghdad Iraq; ^4^ Department of Histology and Cytology, Faculty of Veterinary Medicine Zagazig University Zagazig Egypt; ^5^ Faculty of Veterinary Medicine Hokkaido University Sapporo Japan; ^6^ Internal Medicine Department, Faculty of Medicine Zagazig University Zagazig Egypt; ^7^ University Centre for Research & Development, Chandigarh University Mohali Punjab India; ^8^ Department of Research & Development Funogen Athens Greece; ^9^ Department of Research & Development AFNP Med Wien Austria; ^10^ Department of Science and Engineering Novel Global Community Educational Foundation Hebersham New South Wales Australia; ^11^ Department of Surgery II University Hospital Witten‐Herdecke Wuppertal Germany; ^12^ Department of Biochemistry College of Science University of Jeddah Jeddah Saudi Arabia; ^13^ Department of Pharmacology and Therapeutics, Faculty of Veterinary Medicine Damanhur University Damanhur AlBeheira Egypt

**Keywords:** β‐cells, autophagyinsulin resistancetype 2 diabetes mellitus

## Abstract

Type 2 diabetes (T2D) is a chronic metabolic disorder caused by defective insulin signaling, insulin resistance, and impairment of insulin secretion. Autophagy is a conserved lysosomal‐dependent catabolic cellular pathway involved in the pathogenesis of T2D and its complications. Basal autophagy regulates pancreatic β‐cell function by enhancing insulin release and peripheral insulin sensitivity. Therefore, defective autophagy is associated with impairment of pancreatic β‐cell function and the development of insulin rersistance (IR). However, over‐activated autophagy increases apoptosis of pancreatic β‐cells leading to pancreatic β‐cell dysfunction. Hence, autophagy plays a double‐edged sword role in T2D. Therefore, the use of autophagy modulators including inhibitors and activators may affect the pathogenesis of T2D. Hence, this review aims to clarify the potential role of autophagy inhibitors and activators in T2D.

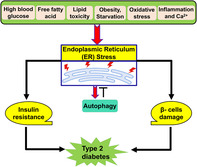

## Graphical abstract

Highlights

- Basal autophagy provides protection against the development of pancreatic β‐cell dysfunction under normal physiological conditions.
- Over‐activated autophagy induces the progression of type 2 diabetes by induction of pancreatic β‐cell apoptosis.
- Autophagy modulators (activators or inhibitors) seem to have either protective or detrimental effects on pancreatic β‐cells.
- Preclinical and clinical studies are warranted using autophagy modulators to manage type 2 diabetes patients.

## 1 INTRODUCTION

**FIGURE 1:**
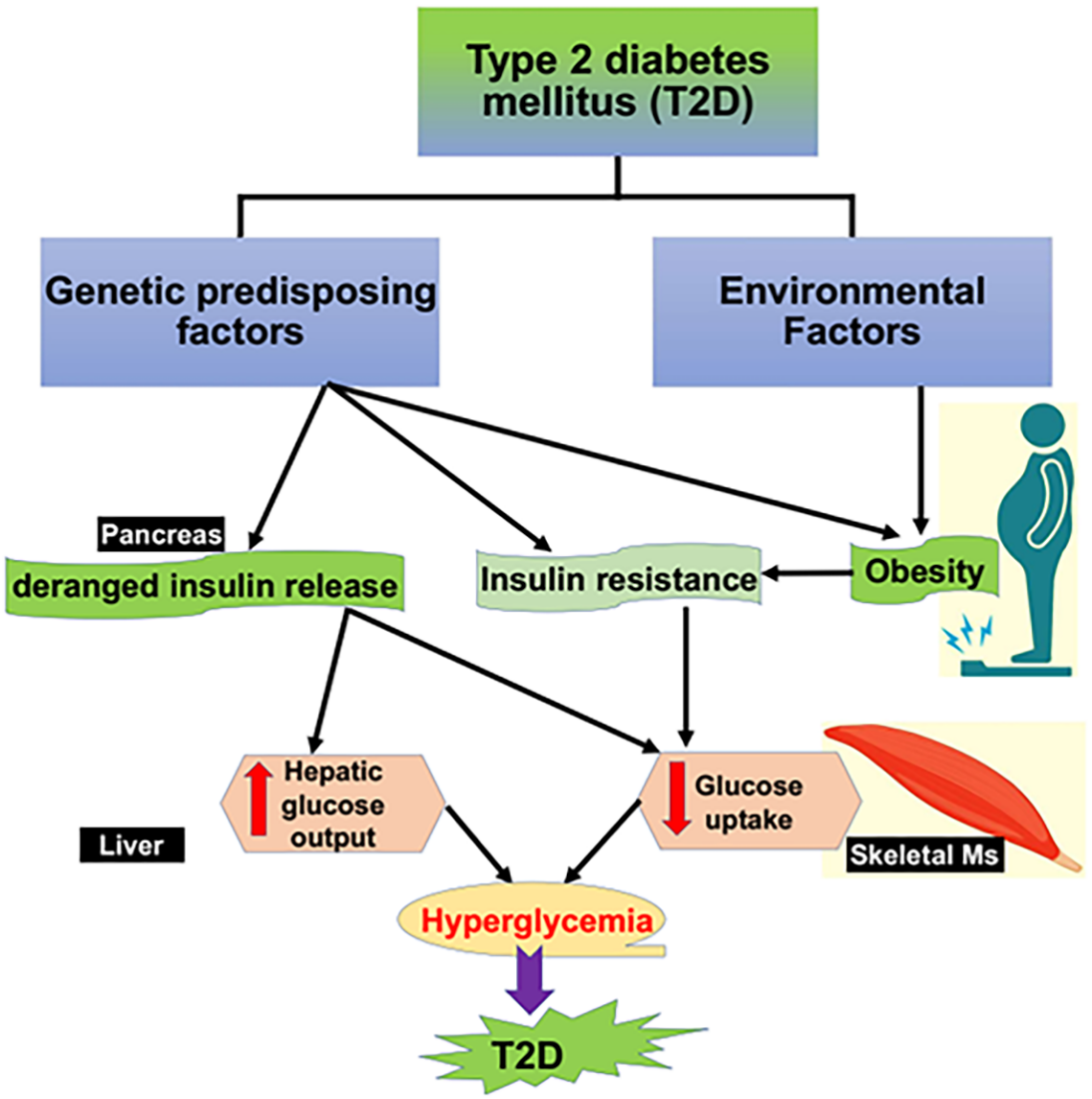
Pathophysiology of type 2 diabetes (T2D): Genetic and environmental factors induce the development of obesity, insulin resistance, and reduce insulin release, causing reduction in peripheral muscle glucose uptake and increasing of hepatic glucose output. These metabolic changes lead to chronic hyperglycemia and the development of T2D.

Type 2 diabetes (T2D) is one of the most common metabolic disorders primarily caused by a combination of two main factors; defective insulin secretion by pancreatic β‐cells and the failure of insulin‐sensitive tissues to respond to insulin.^1^ T2D is the third leading cause of death worldwide and represents 90% of 537 million diabetes cases worldwide.^2^ Of note, 50% of the population older than 65 years, which forms 40% of the general population, has a certain degree of glucose intolerance.^2^ African Americans are more vulnerable to the development of T2D.^3^ It has been shown that T2D is often associated with low‐grade inflammatory disorders due to hyperglycemia‐mediated oxidative stress and the release of proinflammatory cytokines.^4^ Furthermore, environmental and genetic factors are involved with the initiation of chronic inflammation, insulin rersistance (IR), and the development of hyperglycemia in T2D^5^ (Figure 1).

Moreover, glucolipotoxicity and inflammatory cytokines contribute in the development of pancreatic β‐cell dysfunction through induction of endoplasmic reticulum (ER) stress.^6^ In response to these pathological changes in the pancreatic β‐cell dysfunction, autophagy pathway is activated^7^ which is the major cellular pathway for elimination of misfolded proteins and damaged organelles.^8^ Autophagy is a conserved and evolutionarily lysosomal‐dependent catabolic cellular pathway through which abnormal cytoplasmic components including damaged/injured organelles, lipid droplets, and protein aggregates are degraded, and their constituents recycled.^9^ Different signaling proteins involved in the regulation of autophagy flux; for example, Beclin 1, which is a critical component of the class III PI3 kinase complex (PI3KCIII), can induce the formation of autophagosomes.^10^ Triggers of autophagy activate the upregulation of Beclin 1 which promotes PI3KCIII to form Beclin 1–PI3KCIII complex. Therefore, Beclin 1 is required for autophagy differentiation and activation, and depletion of Beclin 1 inhibit autophagy and activate apoptosis.^10^

Moreover, formation of autophagosomes required autophagy‐related genes (ATG) which produce six functional protein groups, including the ATG1 (ULK in mammals) kinase complex, the ATG9 vesicle, the ATG14‐containing PtdIns 3‐kinase complex, the ATG2–ATG18 complex, the ATG12 conjugation system, and the ATG8 conjugation system.^11^ Mammalian ATG8 homologs are called microtubule‐associated protein 1 light chain 3 (LC3) and gamma‐aminobutyric acid receptor–associated protein (GABARAP), which are collectively referred to as ATG8 (s). The ATG8 and ATG12 systems constitute ubiquitin‐like covalent conjugation systems. In the ATG12 system, the most C‐terminal glycine of the ubiquitin‐like protein ATG12 is activated by ATG7, an E1‐like enzyme, in an ATP‐dependent manner, and then sequentially forms thioester intermediates with ATG7 and the E2‐like enzyme ATG10. Finally, ATG12 is conjugated to the acceptor lysine residue in ATG5 via an isopeptide bond. Two sets of ATG12–ATG5 conjugates form a complex with the ATG16(L) dimer. In the ATG8 system, ATG8, another ubiquitin‐like protein, is first synthesized as a proform, whose C‐terminal region is cleaved by ATG4 family enzymes to expose a glycine residue. This processed ATG8 is activated by ATG7 (shared with ATG12), transferred to its specific E2‐like enzyme ATG3, and conjugated to the head group of phosphatidyl ethanolamine (PE). ATG8–PE is present on autophagic membranes.^12^ In contrast to the irreversible ATG12 conjugation, ATG8–PE can be deconjugated again by ATG4. Of note, the ATG12–ATG5 conjugate acts as an E3‐like enzyme to promote ATG8–PE conjugation, which is mediated by an interaction between ATG12 and ATG3. Although ATG16 is not required for the lipidation reaction of ATG8, the membrane binding of ATG16L1 determines the site of ATG8 lipidation. ATG8 lipidation could occur on nonautophagic membranes, but it is efficiently corrected by ATG4‐mediated deconjugation that can be regulated by ATG1 and ULK1. The processed unlipidated ATG8 and ATG8–PE are called ATG8‐I and ATG8‐II, respectively (e.g., LC3‐I and LC3‐II). ATG8 is the most commonly used autophagosome marker, and ATG5 and ATG7 have been frequently used in knockout mouse studies.^11^, ^12^

Therefore, autophagy plays a crucial role in maintaining and preserving of cellular homeostasis in response to the different intracellular stressors.^9^ However, efficiency and functional activity of autophagy process is highly reduced during aging and by over‐nutrition which interferes with autophagic flux.^9^ As well, obesity, IR, T2D, and other metabolic disorders which are linked with aging are often associated with increment of intracellular stress result in more deterioration of cellular homeostasis. Aging‐mediated autophagy dysfunction further aggravates IR and T2D.^9^, ^13^ In addition, autophagy contributes in cellular nutrition during starvation, improves pancreatic β‐cells, and increases peripheral insulin sensitivity.^13^ Indeed, basal autophagy and normal autophagy response are regulated by various intracellular nutrient‐sensing pathways including AMP–activated protein kinase (AMPK), mechanistic target of rapamycin complex 1 (mTORC1), and sirtuin 1 (SIRT1).^14^ Both AMPK and SIRT1 activate autophagy, whereas mTORC1 inhibits autophagy under physiological conditions. For example, caloric restriction which increases the expression of AMPK and SIRT1, improves autophagy process with subsequent reduction of IR and T2D development.^15^ However, aberrant over‐expression of mTORC1 induces metabolic disorders by inhibiting autophagy flux.^15^ Basal autophagy under normal physiological conditions seems to be protective against the development of pancreatic β‐cell dysfunction.^14^ Conversely, over‐activated autophagy increases apoptosis of pancreatic β‐cells.^15^ Thus, autophagy plays a double‐edged sword role in T2D.^16^ Therefore, use of autophagy modulators including inhibitors and activators may affect the pathogenesis of T2D.^17^ Therefore, this review aims to clarify the potential role of autophagy inhibitors and activators in T2D.

## 2 AUTOPHAGY IN T2D

**FIGURE 2:**
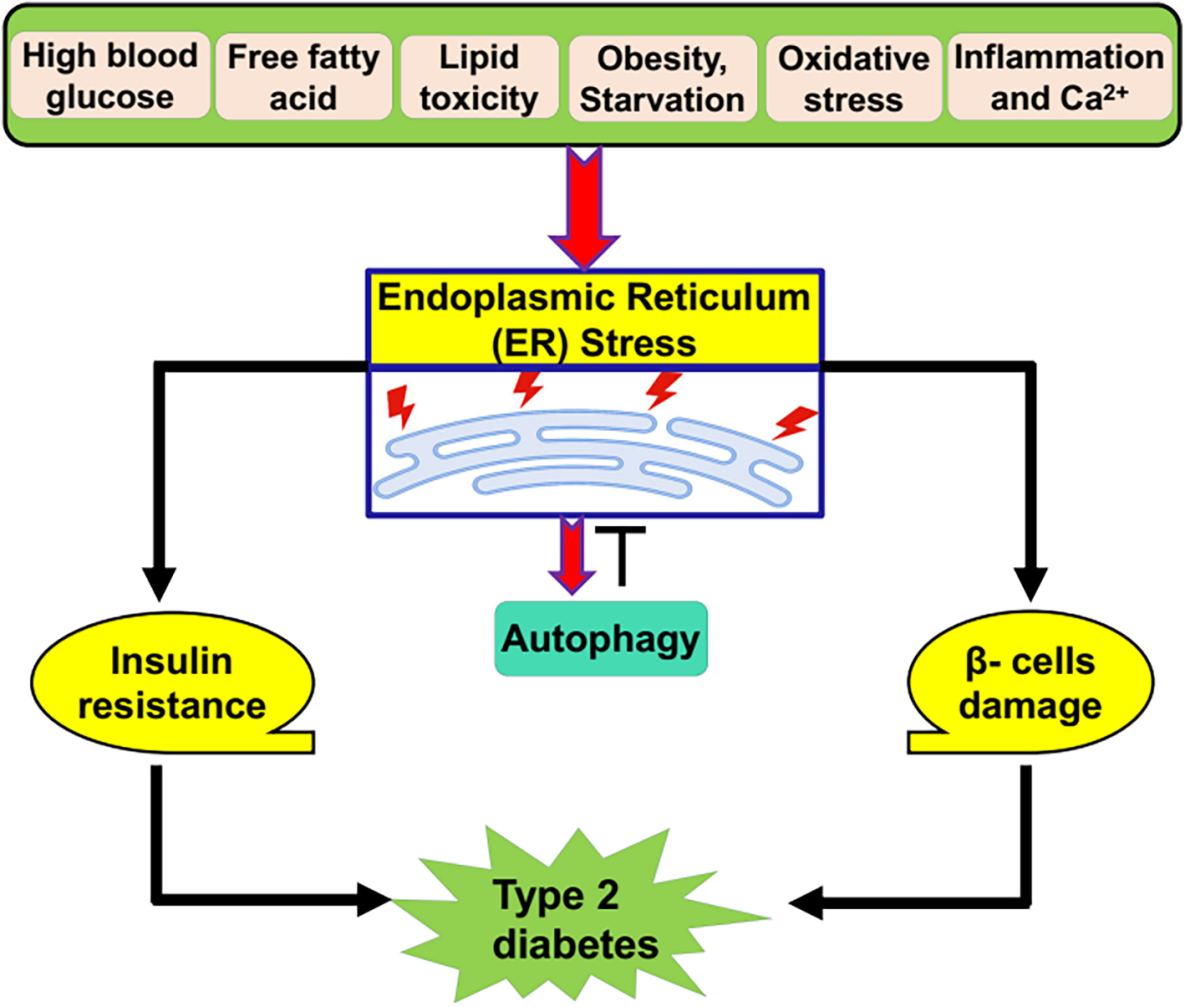
ER stress and autophagy in type 2 diabetes (T2D): High blood glucose and fatty acid, and associated obesity, oxidative stress, and inflammation induce the development of endoplasmic reticulum (ER) stress, which affect autophagy function, induce pancreatic β‐cell damage, and the development of insulin resistance. These changes contribute in the development of T2D.

Hyperglycemia, glucolipotoxicity, ER stress, oxidative stress, inflammation, and Ca^2+^ dyshomeostasis contribute to the development of pancreatic β‐cell dysfunction, IR, and the development of T2D.^18^, ^19^, ^20^, ^21^, ^22^, ^23^ Prolong ER stress triggers activation of autophagy, which also inhibits ER stress^24^ (Figure 2).

### 2.1 Beneficial effects of autophagy in T2D

Basal autophagy preserves and protects pancreatic β‐cell function from the effect of oxidative stress.^25^ Interestingly, dysregulated autophagy represents a key player in the pathophysiology of T2D and its complications. Basal autophagy promotes insulin signaling in both pancreatic β‐cells and peripheral tissues.^25^ Thus, age‐mediated defective autophagy is implicated in the development of T2D and associated macrovascular and microvascular complications.^25^

**FIGURE 3:**
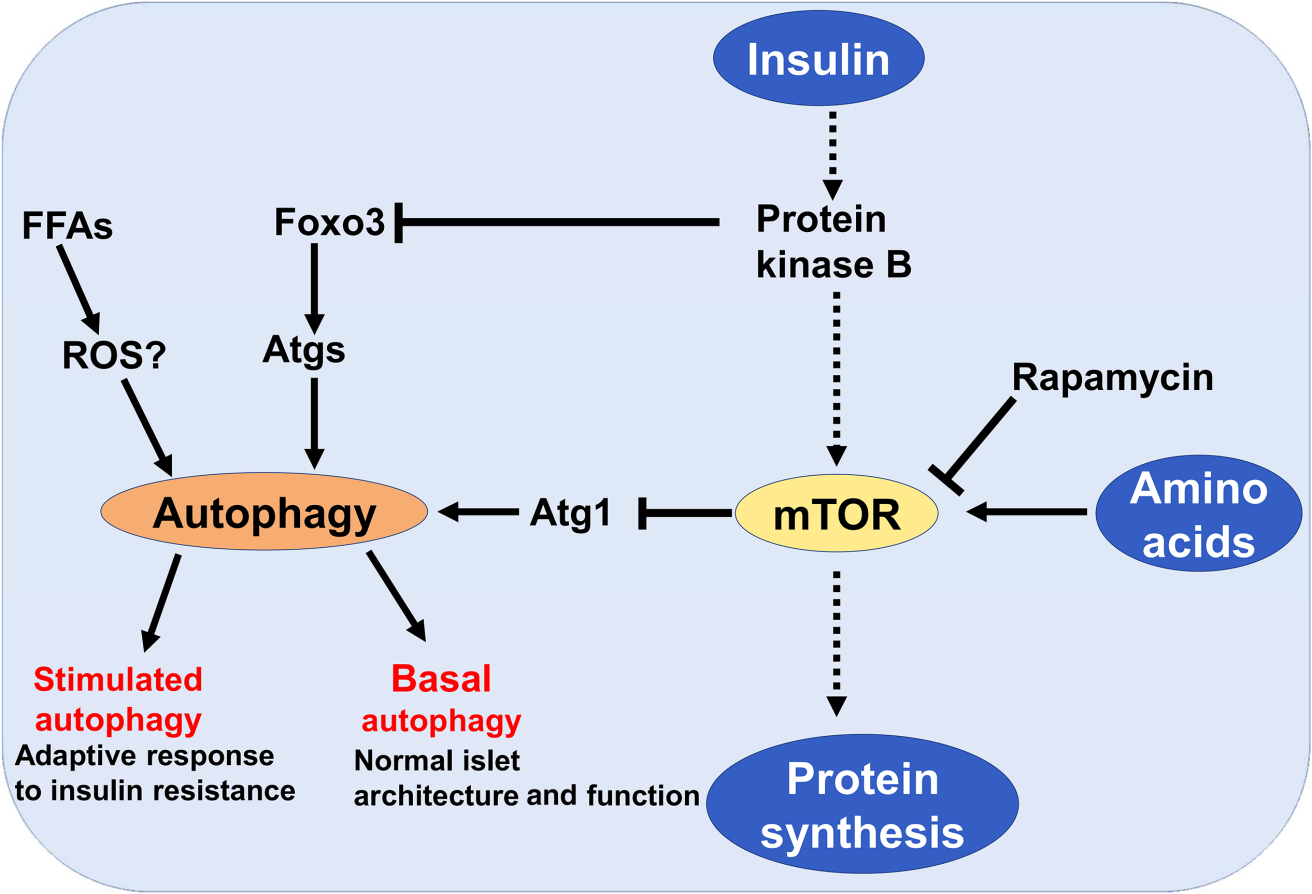
Insulin and autophagy in type 2 diabetes (T2D): Insulin through activation of protein kinase B inhibits autophagy either directly by inhibiting of forkhead box O3 (Foxo3) signaling or indirectly through activation of mammalian target of rapamycin (mTOR), which inhibit autophagy through downregulating of autophagy‐related gene 1 (Atg1). Amino acids and rapamycin activate and inhibit mTOR, respectively.

In a state of IR, hyperinsulinemia inhibits autophagy by activating mTOR in synergy with amino acids leading to the inhibition of autophagy‐related gene 1 (Atg1) which involved in the activation of autophagy.^26^, ^27^, ^28^, ^29^ Insulin‐mediated activation of protein kinase B also inhibit forkhead box O3 (FOXO3) which activates autophagy^30^ (Figure 3).

**FIGURE 4:**
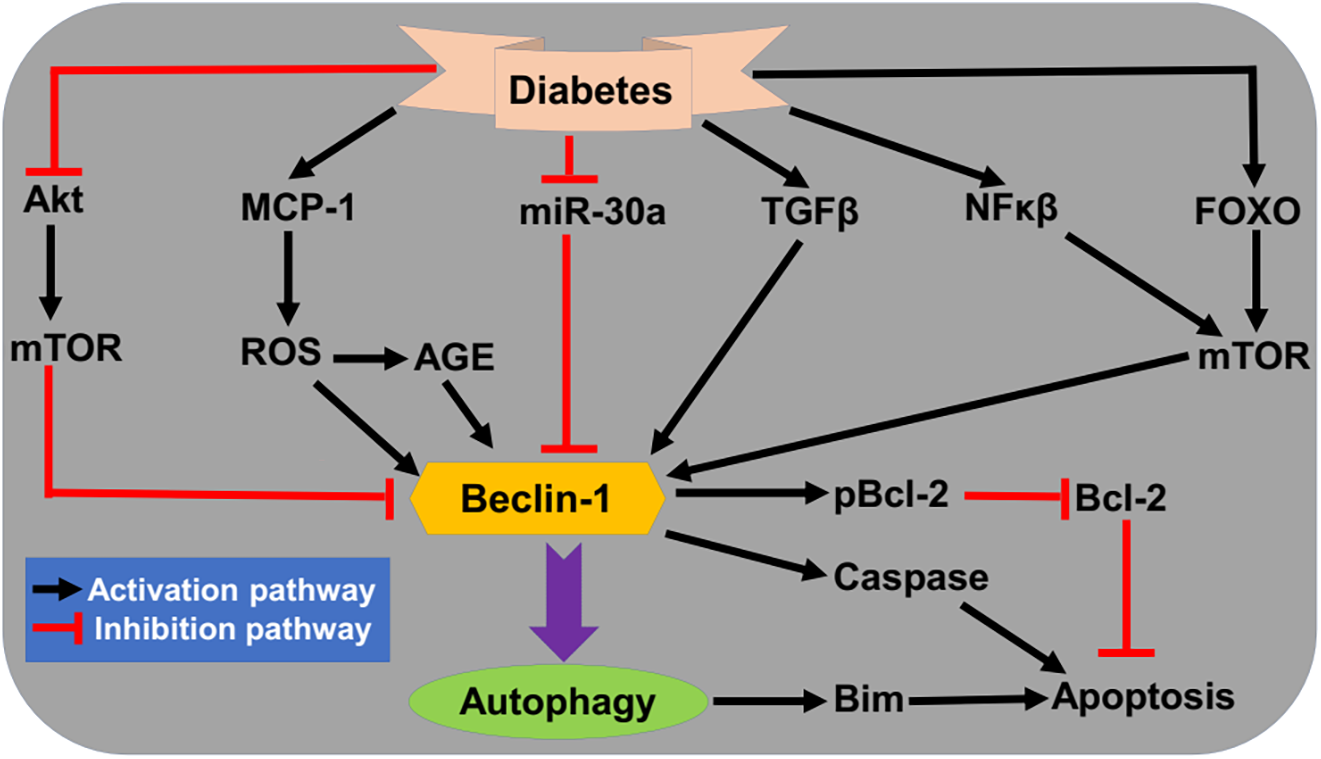
Activated autophagy in type 2 diabetes (T2D): Diabetes causes the activation and inhibition of many signaling pathways that affect the activation of autophagy through Beclin‐1‐dependent pathway. Metabolic alterations in diabetes activate monocyte chemoattractant protein 1 (MCP‐1), tumor growth factor beta (TGF‐β), nuclear factor kappa B (NF‐κB), and Foxo. However, Akt and miR‐30a which inhibit autophagy or through mammalian target of rapamycin (mTOR) pathway are inhibited in diabetes. These changes alter the functional activity of autophagy in T2D. miR‐30a, microRNA 30a; Akt, protein kinase B.

In T2D, different signaling pathways are dysregulated which affect expression of Beclin‐1.^31^ For example, tumor growth factor beta (TGF‐β), nuclear factor kappa B (NF‐κB), and transcription factor FOXO3 activated Beclin‐1. Besides, reactive oxygen species (ROS) and advanced glycation end‐product (AGE) in response to the activated monocyte chemoattractant protein 1 (MCP‐1) induces Beclin‐1. However, suppression of Akt and mTOR by diabetes blocks the function of Beclin‐1.^31^ Autophagy activation in T2D in response to different signaling pathways may be a compensatory pathway to overcome inflammatory and oxidative disorders^30^ (Figure 4).

During IR development, autophagy is increased in the pancreatic β‐cells as a compensatory mechanism to overcome oxidative and inflammatory disorders.^32^ Genetic deletion of Atg7 in mice triggers degeneration of pancreatic β cell, inhibition of insulin secretion, induction of abnormal glucose intolerance, and development of diabetes.^33^ Loss of autophagy promotes the accumulation of ubiquitin‐containing proteins and proteins expressing LC3‐binding protein p62 which is required for delivery of aggregated proteins to autophagosomes.^34^, ^35^, ^36^ In addition, loss of autophagy induce the accumulation of distended ER and malformed mitochondria.^33^ It has been shown that autophagy‐deficient mice experience hyperglycemia and hyperinsulinemia due to the development of mitochondrial dysfunction and ER stress.^37^ Notably, Atg7 mutant mice showed augmented apoptosis and reduced proliferation of pancreatic β‐cells leading to significant decrease of β‐cell mass.^37^ As well, glucose induced Ca^2+^ signaling which is necessary for insulin release is severely impaired in Atg7 mutant mice.^37^ Defective autophagy promotes the development of ER stress and mitochondrial dysfunction leading to dysfunction of pancreatic β‐cells through defect in ATP formation and augmentation of oxidative stress.^38^ Findings from clinical studies observed extensive accumulation of autophagy vacuoles and p62 in pancreatic β‐cells of T2D patients^39^, ^40^ suggesting defective autophagy flux in T2D. These findings indicated that basal and even constitutive autophagy is essential for homeostasis of pancreatic β‐cells.^41^

High‐fat diet increases level of autophagosomes in both diabetic and non‐diabetic mice due to impairment the interaction between autophagosomes and lysosomes or failure of lysosomal proton pump.^42^ When Atg^−/−^ mice fed on high fat diet, their blood glucose tolerance were further deteriorated, indicating importance of autophagy in the regulation function of pancreatic β‐cells.^42^ It has been illustrated that pancreatic β‐cell mass is reduced by increasing apoptosis in Atg7‐deficient mice. Besides, in vitro study demonstrated that coadministration of lysosomal inhibitors and fatty acids increase autophagy flux as evident by increasing of LC3‐II in mice.^42^ These observations proposed that autophagy increases IR to protect pancreatic β‐cells against oxidative injury. In addition, IR‐induced autophagy is regarded as an adaptive response to prevent glucolipotoxicity.^42^

In addition, obesity‐induced IR is mediated by leptin resistance which affects the expression of autophagy genes leading to the inhibition of autophagy flux and impairment of autophagy.^43^ Goldstein et al.^43^ illustrated that leptin improves autophagy and lysosomal related degradation of misfolded proteins in adipocytes.

Furthermore, autophagy plays a critical role in preventing the accumulation of islet amyloid polypeptide (IAPP) in only human pancreatic β‐cells, but not in rodents due to the absence of IAPP expression in rodents.^22^ Progressive accumulation of IAPP in transgenic mice expressing human IAPP are susceptible to developing diabetes.^44^ Autophagy increases cellular elimination of IAPP thereby attenuating failure of pancreatic β‐cells and the development of T2D. Therefore, activation of autophagy could be effective in preventing the development of neurodegenerative diseases including Alzheimer disease in T2D patients.^22^

These findings indicated that activated autophagy in T2D has a protective effect to prevent further deterioration of pancreatic β‐cells.

### 2.2 Detrimental effects of autophagy in T2D

On the other hand, different studies reported that autophagy plays a detrimental role in the pathogenesis of T2D.^45^, ^46^, ^47^ The harmful effect of autophagy is related to the induction of ROS accumulation through Atg5‐dependent pathway and accelerating of pancreatic β‐cell deaths. Moreover, autophagy can induce cell deaths by inhibiting caspase pathway through induction of ROS formation, oxidation of lipid membrane and injury of plasma membrane.^47^ It has been shown that knocking of Atg7 or Atg8 and use of autophagy inhibitors may attenuate ROS‐induced cell deaths.^47^ Autophagic cell death occurs through the interaction of Atg5 with Fas‐associated protein with death domain (FADD).^45^ Interferon gamma (INF‐γ)‐induced autophagic cell death is mediated by the expression of Atg5.^45^ Furthermore, Atg5 promotes apoptotic stimuli in cancer cells both in vitro and in vivo.^46^ Likewise, a Ca^2+^‐dependent nonlysosomal cysteine protease calpain, which is expressed ubiquitously, has ability to induce apoptosis through activation of Atg5.^46^ Genetic variation in calpain‐like cysteine, calpain 10 is associated with pancreatic β‐cell deaths.^48^ Of note, calpain pathway is augmented leading to platelet activation and thrombosis in T2D patients.^49^ Therefore, exaggerated calpain pathway in T2D may associate with progressive pancreatic β‐cell deaths via activation of apoptosis and autophagic cell death.

Furthermore, mTOR a known inhibitor of autophagy plays an important in preventing oxidative stress‐induced pancreatic β‐cell deaths mediated by apoptosis and autophagy. Inhibition of apoptotic pathways by mTOR limits the development of IR and T2D due to pancreatic β‐cell deaths.^50^ In addition, GLP‐1 agonists protect pancreatic β‐cells by activating mTOR signaling.^51^ Findings from experimental studies highlighted that autophagy may induce progressive death of pancreatic β‐cells. In addition, induced autophagy promotes loss of hepatic and cardiac tissues in diabetic rats.^52^

These findings proposed that autophagy plays a detrimental role on the pancreatic β‐cells leading to acceleration of cell deaths and development of T2D.

Taken together, autophagy has a double edge‐sword that could be beneficial or harmful in T2D. Therefore, use of autophagy modulators is logical in this regard to clarify the beneficial effects of autophagy activators or inhibitors in T2D.

## 3 AUTOPHAGY MODULATORS

Formation of autophagosomes is mediated by Atg1/PI3K, Atg8, and At5–Atg12 conjugation systems.^53^ Beclin‐1 through interaction with class III PI3K initiates the formation of autophagosomes.^54^ Notoriously, mTOR plays a central player in the regulation of autophagy signaling in response to starvation and hypoxia.^55^ Moreover, calpain, PI3K, cAMP, and Ca^2+^ are also involved in the regulation of autophagy signaling.^56^ In addition, histone deacetylase 6 (HDAC6) regulates the interaction between autophagosomes and lysosomes.^57^ Therefore, targeting of these signaling mainly Beclin‐1, mTOR, calpain, PI3K, cAMP, HDAC6, and Ca^2^ by specific modulators could be an effective therapeutic strategy in the management of T2D.

### 3.1 Autophagy activators in T2D

It has been shown that activation of autophagy by caloric restriction promotes regeneration of pancreatic β‐cells. This effect mimic the effect produced by using mTORC1 inhibitors signifying that autophagy is an essential pathway for homeostasis of pancreatic β‐cells.^58^ In addition, autophagy activators promote neurogenesis of pancreatic β‐cells and prevent their apoptosis. As well, autophagy augments peripheral insulin sensitivity mainly in the liver and skeletal muscles.^58^, ^59^ Remarkably, many antidiabetic drugs such as rosiglitazone, metformin and glucagon like peptide 1 (GLP‐1) prevent dysfunction of pancreatic β‐cells by inducing autophagy by increasing the expression of AMPK which induce autophagy by inhibiting mTORC1 or though activation of Vps34 a complex activated by Beclin‐1.^59^ Therefore, autophagy activators are involved in the management of T2D.

#### 3.1.1 mTOR inhibitors

**FIGURE 5:**
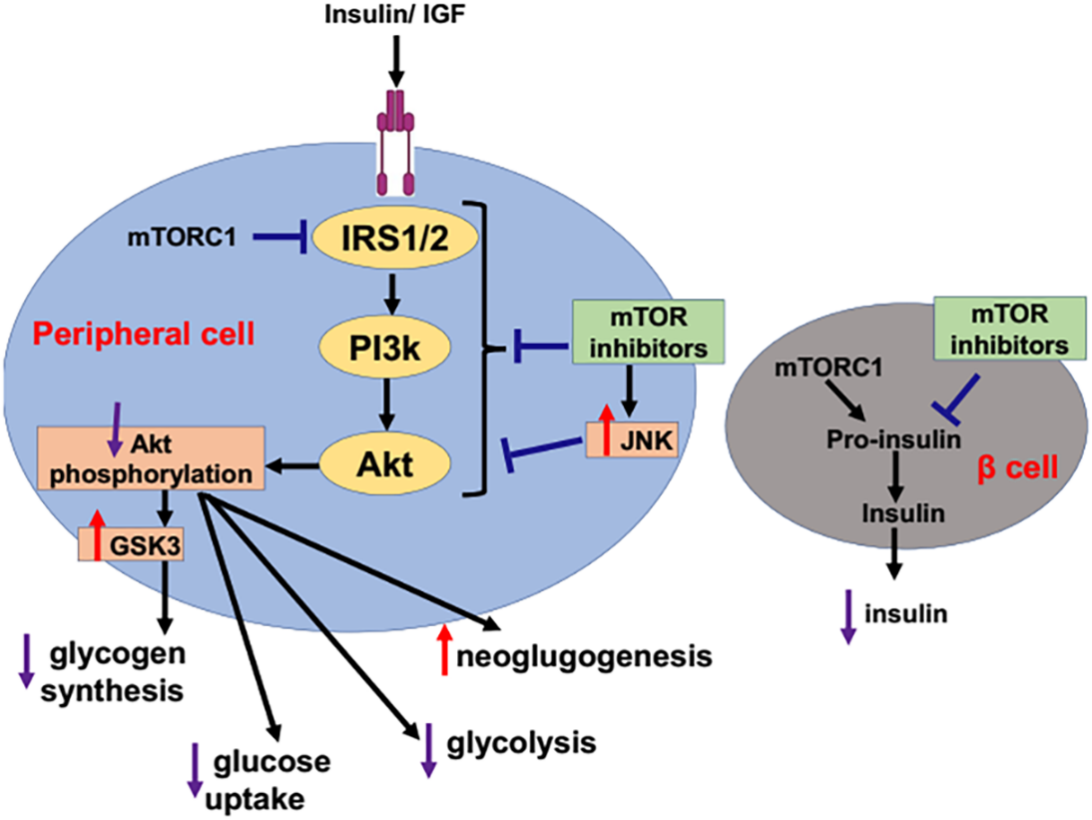
mTOR inhibitors and risk of type 2 diabetes (T2D): Mammalian target of rapamycin (mTOR) inhibitors reduce the conversion of proinsulin to insulin in the pancreatic β‐cells that increase risk of T2D development. In addition, mTOR inhibitors by increasing JNK signaling attenuate the activation of insulin receptor substrate 1/2 and P13k/Akt signaling activation leading to alteration of glucose metabolism. Decreasing Akt phosphorylation activates glycogen synthase kinase 3 (GSK3), which inhibits glycogen synthesis. Inhibition of Akt phosphorylation leads to activation of gluconeogenesis and inhibition of glycolysis and glucose uptake. IGF, Notice insulin‐like growth factor; IRS1/2, Insulin receptor substrates 1/2; mTOR, the mammalian target of rapamycin (mTOR); mTORC1, mammalian target of rapamycin complex1; PI3Ks, Phosphatidylinositol 3‐kinases; Akt, protein kinase B; GSK3, Glycogen synthase kinase‐3; JNK, Jun N‐terminal kinase.

Rapamycin which also termed sirolimus is a potent immunosuppressive and antifungal natural product.^60^ Rapamycin interacts with immunophilin to form a complex which inhibits the kinase activity of mTOR leading to induction of autophagy.^60^ In addition, ester of rapamycin which called temsirolimus has ability to induce autophagy.^61^ Of note, mTOR inhibitors are primarily used as immunosuppressive agents in the management of various cancer types including renal cell carcinoma, breast cancer, and neuroendocrine tumors.^62^ mTOR signaling plays a critical role in glucose metabolism and homeostasis. It has been reported that early use of mTOR inhibitors is associated with the development of new‐onset T2D.^63^, ^64^, ^65^, ^66^ mTOR inhibitors‐induced hyperglycemia is mediated by the development of IR and impairment of insulin secretion.^62^, ^67^ Fraenkel et al.^67^ observed that mTOR inhibitor rapamycin attenuates adaptation of pancreatic β‐cells to the effect of hyperglycemia and contribute to the exacerbation of metabolic complications in T2D (Figure 5).

Rapamycin can cause fulminant T2D in experimental animals by reducing mTOR signaling which is essential for the function of pancreatic β‐cells.^67^ It has been shown that mTOR signaling triggers the activation of ribosomal S6 kinase 1 (S6K1) and 4 eukaryotic binding protein 1 (4EBP1).^68^, ^69^, ^70^ Insulin activates mTOR signaling via PI3K/Akt pathway. In turn, mTOR/S6K1 signaling induces negative feedback inhibition of insulin sensitivity.^71^ Thus, over‐activation of mTOR/S6K1 signaling is associated with impairment of insulin action.^72^ Similarly, stimulation of mTOR/S6K1 signaling by amino acid induces IR which prevented by rapamycin.^73^ Therefore, mTOR inhibitor rapamycin could be effective in the regulation of glucose metabolism and insulin sensitivity in mice.^74^ An experimental study demonstrated that rapamycin improves hepatic insulin sensitivity and inhibit autophagy in rats with hepatic steatosis and IR.^75^ A recent experimental study conducted by Reifsnyder et al^76^ illustrated that co‐treatment with metformin and rapamycin improves insulin sensitivity in mice. Puzzling, rapamycin has a dual role it can induce IR and at the same time improves insulin sensitivity. Also, rapamycin can cause glucose intolerance without IR. This effect of rapamycin is called benevolent pseudo‐diabetes.^77^ Conversely, rapamycin enhances life extension without diabetogenic effect in healthy subjects when used with caloric restriction and metformin.^77^, ^78^ Furthermore, insulin sensitizing agent metformin which used in the management of T2D has ability to blocks mTOR pathway independent of AMPK pathway.^79^ Findings from in vitro and in vivo studies unveiled that metformin has anticancer effect via inhibition of mTOR pathway.^80^ Interestingly, different studies revealed that metformin can induce autophagy through activation of AMPK and inhibition of mTOR pathway.^81^, ^82^ Furthermore, an isoquinoline alkaloid berberine has anti‐inflammatory effects by inducing autophagy through inhibition of mTOR pathway.^83^ Pang et al.^84^ illustrated that berberine is effective in the management of T2D by different mechanisms including inhibition of mTOR pathway and activation of autophagy pathway.

Therefore, mTOR inhibitors rapamycin, metformin, and berberine through induction of autophagy improve glucose homeostasis and prevent T2D‐induced complications.

#### 3.1.2 ER stressing inducers

ER stress is regarded as a potent autophagy inducer. Notably, Sar1 and Rab1b are GTPase required for formation of autophagosomes.^85^ ER stress promotes autophagy by inhibition of mTOR/Akt pathway.^85^ ER stress inducers like thapsigargin, brefeldin, and tunicamycin enhance the expression of autophagic vesicles and Beclin‐1.^86^

Thapsigargin is an inhibitor of ER Ca^2+^ ATPase (SERCA) extracted from *Thapsia garganica*.^87^ Thapsigargin increases cytosolic Ca^2+^ by inhibiting Ca^2+^ pump into ER and sarcoplasmic reticulum.^88^ Depletion of Ca2^+^ within ER and sarcoplasmic reticulum trigger the opening of plasma membrane Ca^2+^ channel.^89^ This effect induces ER stress and expression of unfolded protein response (UPR) to overcome ER stress.^90^ However, unresolved ER stress triggers apoptosis and cell death. Besides, Ca^2+^ store depletion prevents ferroptosis through phospholipids from ER.^91^ Thapsigargin via induction of ER stress activates autophagy.^92^ It has been shown that thapsigargin triggers apoptosis of pancreatic β‐cells.^93^ Inhibition of SERCA reduces functional activity of pancreatic β‐cells and insulin release.^94^

It has been shown that alteration of cytosolic Ca^2+^ is linked with development of T2D.^95^ In vitro study demonstrated that lymphocytes from T2D patients had higher cytosolic Ca^2+^ and correlated with blood glucose as compared to controls.^95^ Therefore, ER stress inducers may aggravate cytosolic Ca^2+^ by enhancing Ca^2+^ flux across the cell membrane. In addition, ER stress inducers reduce the expression of superoxide dismutase causing oxidative stress and exacerbation of T2D.^96^ However, ER stress inducers promote the expression of UPR which has a protective effect against T2D.^90^, ^94^ Notoriously, augmentation of UPR by ER stress inducers is involved in the attenuation of pancreatic β‐cells death.^97^ UPR conserves ER homeostasis and attenuates β‐cell failure. However, prolong ER stress reduces the protective effects of UPR leading to β‐cell death and reduced insulin secretion.^98^, ^99^ Therefore, ER stress inducers have a protective effect by increasing UPR and detrimental effect by inducing oxidative stress in T2D regardless of autophagy.

#### 3.1.3 Inositol monophosphatase inhibitors

Inositol monophosphatase (IMPase) is an enzyme involved in the regulation of intracellular IP3 and free inositol. Different types of IMPase inhibitors include lithium, valproic acid, carbamazepine and L‐690330.^100^ IMPase inhibitors induce autophagy in mTOR‐independent pathway and can be used in neurodegenerative diseases.^101^ IMPase inhibitor lithium has a critical role in treating neurodegenerative diseases by enhancing autophagy.^102^ It has been shown that lithium reduced T2D risk by inhibition of glycogen synthase kinase 3.^103^ A retrospective study illustrated that long‐term use of lithium decreases T2D risk.^103^ Of note, neuroprotective agents like lithium have cytoprotective effects against injury of pancreatic β‐cells.^104^ Experimental study indicated that a microdose of lithium protects pancreatic β‐cells.^105^ In addition, antiepileptic carbamazepine enhances survival of pancreatic β‐cells.^106^ Preclinical study observed that carbamazepine slows the progression of pancreatic β‐cell injury by reducing inflammation in mice.^106^ Furthermore, antiepileptic valproic acid has the ability to decrease IR, gluconeogenesis, and fat deposition by inhibiting IMPase and induce autophagy.^107^ The underlying mechanism for the beneficial effect of valproic acid on glucose homeostasis and T2D is related to the modulation of HDAC, insulin signaling, glucagon secretion, and expression of FOXO1.^107^ Of note, HDAC signaling which augments IR is increased in T2D patients.^108^ Therefore, use of HDAC inhibitors like valproic acid could be effective in attenuating the development of IR and T2D.^108^ In addition, valproic acid, carbamazepine, and lithium have been to induce autophagy.^109^, ^110^, ^111^ Therefore, IMPase inhibitors seem to be effective in T2D by inducing autophagy.

#### 3.1.4 Trehalose

Trehalose is a nonreducing sugar consisting of two glucose unites fused by alpha bond named α‐D‐glucopyranosyl.^112^ Trehalose is derived from rye ergot that reduces protein denaturation and protect cell membrane integrity.^113^ Trehalose via induction of autophagy increases the elimination of α‐synuclein and can be used in the management of neurodegenerative diseases.^114^ Trehalose is not synthesized inside human body; it is commonly used as food stabilizer.^115^ Ingested trehalose is metabolized by trehalases; therefore, trehalose is regarded as landmark of carbohydrate metabolite, and high blood trehalose level is associated with risk for the development of T2D.^115^ It has been shown that trehalose can reduce the development of T2D by antioxidant effect in experimental animals.^116^ Korolenko et al.,^117^ observed that trehalose induces myocardial and hepatic autophagy in diabetic mice. A placebo controlled clinical trial on 34 subjects with high body mass index showed that daily intake of trehalose 10 g per day for 12 weeks reduced IR and improved blood glucose homeostasis.^118^ This finding suggests that prolonged intake of trehalose attenuates the development of IR and metabolic syndrome in high‐risk patients. A case control study involved 69 pairs of diabetic retinopathies and matched T2D patients showed that treatment with trehalose decreased risk of diabetic retinopathy.^119^ In addition, trehalose prevents IR and postprandial insulin burst.^120^ These observations indicated that trehalose reduces T2D risk by inducing autophagy and antioxidant effects.

#### 3.1.5 PI3K inhibitors

PI3K pathway is intricate in different cellular functions including cell growth, differentiation, proliferation, and inhibition of autophagy.^121^, ^122^, ^123^, ^124^, ^125^ Therefore, PI3K inhibitors like ceramide increase the expression of Beclin‐1 and improve autophagy function.^126^ Sphingosine‐1‐phosphate, like ceramide, promotes autophagy in many cancer cell lines.^127^ Ceramide regulates various cellular signaling including apoptosis and cell‐cycle arrest. Ceramide is phosphorylated to ceramide‐1 phosphate which has opposite effect and can induce inflammatory reactions via stimulation of cytosolic phospholipase A2 and consequent release of prostaglandins.^128^, ^129^, ^130^, ^131^ High ceramide is associated with the development of IR.^132^ Of note, ceramide inhibits insulin action by attenuating the expression of Akt/protein kinase B.^133^ Therefore, progressive tissue accumulation of ceramide participates in the development and progression of IR.^133^, ^134^, ^135^, ^136^ Thus, inhibition of ceramide prevents IR and the development of T2D induced by glucocorticoid therapy.^137^ Ceramide has a diabetogenic effect through induction inflammation and apoptosis of pancreatic β‐cells.^138^ Furthermore, increasing tissue ceramide promotes the development and progression of T2D‐related complications (137; 138). Therefore, ceramide seems to be harmful in T2D despite induction of autophagy.

#### 3.1.6 Calpain inhibitors

Calpain is a Ca^2+^‐dependent, nonlysosomal cysteine protease expressed ubiquitously involved in cell‐cycle progression and cell mobility.^139^ Calpain has ability to inhibit autophagy due to high cytosolic Ca^2+^ activates calpain.^140^ Therefore, calpain inhibitor calpastatin could be effective to induce autophagy.^141^ Variation in the expression of calpain‐10 gene in the pancreatic β‐cells increases risk of T2D. Caplain regulates the function of pancreatic β‐cells and improves insulin secretion. Therefore, calpastatin inhibits the functional activity of pancreatic β‐cells and may exacerbate T2D.^142^ Zhu et al.^143^ revealed that caplain over‐expression and calpastatin down‐regulation mediates the development of Alzheimer disease (AD) in diabetic mice. This finding suggests that chronic hyperglycemia‐induced AD via inhibition of calpastatin‐mediated autophagy. Interestingly, calpastatin‐mediated autophagy prevents cardiac fibrosis in murine model of T2D.^144^ Other calpain inhibitors including peptidyl epoxide, ketoamide inhibitors, and aldehyde were shown to be effective against various metabolic diseases including T2D.^145^ Therefore, autophagy induction by calpastatin could be an effective novel therapeutic strategy in preventing misfolded protein‐induced pancreatic β‐cell dysfunction.^146^

#### 3.1.7 Inositol triphosphate inhibitors

Inositol triphosphate (IP3) is cytosolic signaling pathway involved in the regulation of insulin secretion and inhibition of autophagy.^147^ Expression of IP3 receptor in T2D patients is reduced leading to abnormal autophagy function.^148^ IP3 receptors mediate insulin release in response to glucose administration.^149^ IP3 receptor antagonist xestospondin inhibits insulin release in response to glucose.^149^ Of note, xestospondin is regarded as mTOR‐independent autophagy activator.^150^ Therefore, IP3 receptor antagonists improve autophagy but at the same time deteriorate insulin secretion and increase T2D risk.

#### 3.1.8 Ca^2+^ channel blockers

Ca^2+^ is an essential intracellular second messenger regulating various cellular processes including autophagy. High intracellular Ca^2+^ inhibits autophagy in hepatocytes through inhibition the interaction between autophagosomes and lysosomes.^151^ Therefore, Ca^2+^ channel blockers (CCBs) like verapamil can activate autophagy by inducing the interaction between autophagosomes and lysosomes.^152^ CCBs through induction of autophagy prevent the accumulation of lipid droplets and protein inclusions with subsequent inhibition of inflammation and IR.^152^ Herein, CCBs may reduce the metabolic complications in obesity by enhancing autophagy and decreasing the development of IR. It has been reported that CCBs reduce defective autophagy‐induced IR in obesity.^153^ Furthermore, CCBs improve diabetic outcomes and prevent the progression of cognitive deficits and depression.^154^ Remarkably, CCBs inhibit the activation of aldose reductase pathway which transforms glucose to sorbitol leading to the development of diabetic complications.^155^ Among CCBs, cinnarizine is the most potent inhibitor of aldose reductase pathway.^155^ In addition, CCB nifedipine improves glucose homeostasis and lipid profile in T2D patients.^156^ Collectively, CCBs via induction of autophagy may improve glucose homeostasis and reduce diabetic complications.

### 3.2 Autophagy inhibitors in T2D

It has been illustrated that autophagy inhibitors could be effective against the development of IR and T2D by inhibiting pancreatic β‐cell deaths. Autophagy triggers apoptosis by inhibiting caspase pathway and induction of ROS formation, oxidation of lipid membrane, and injury of plasma membrane.^47^ It has been shown that uses of autophagy inhibitors may attenuate ROS‐induced cell deaths.^47^ An updated experimental study revealed that autophagy inhibitors prevent retinal inflammation in diabetic mice.^148^ Autophagy inhibitors can prevent hyperglycemia‐induced exaggeration of autophagy, which is harmful rather than beneficial. Of note, low autophagy is required for normal pancreatic β‐cell function; however, exaggerated autophagy triggers autophagic‐programmed cell death and apoptosis.^47^ Therefore, autophagy inhibitors can mitigate IR‐induced autophagy over‐activity and associated inflammatory and oxidative stress disorders.

#### 3.2.1 Lysosomal alkalizer

Lysosomal lumen alkalizers like chloroquine and hydroxychloroquine are anti‐malarial and anti‐inflammatory agents, which act by impairing of lysosomal function and inhibiting of autophagy.^157^ Chloroquine‐induced autophagy inhibition is mediated by inhibiting the interaction between autophagosomes and lysosomes rather than affecting the acidity or degradative capacity.^158^ A previous clinical trial observed that hydroxychloroquine was effective in the management of T2D refractory to the effect of sulfonylureas.^159^ In comparison with pioglitazone, hydroxychloroquine was effective in the management of T2D through modulation of lipid metabolism and glucose homeostasis.^160^ A systematic review and meta‐analysis of 11 randomized controlled clinical trials showed that there was no strong clinical evidence to recommend use of hydroxychloroquine in the management of T2D.^161^ These findings proposed that hydroxychloroquine may have some beneficial effect in the management of T2D, despite of the inhibitory effect on the autophagy function.

Moreover, lys01 is a dimeric form of two chloroquine moieties that is 10 time more potent autophagy inhibitor compared to hydroxychloroquine.^162^ A water‐soluble form of lys01 is known as lys05 has higher ability to deacidifying the lysosomes and inhibition of autophagy.^163^ However, the effects of lys01 and lys05 were not evaluated on insulin sensitivity and pancreatic β‐cell functions.

#### 3.2.2 Cycloheximide

Cycloheximide is a protein synthesis inhibitor produced by *Streptomyces griseus*. Cycloheximide is widely used in various biomedical research regarding autophagy function. Because of the toxic adverse effects of cycloheximide, including teratogenesis and DNA damage, it used in research only.^164^ It has a potent inhibitor effect on the autophagy function by inhibiting the formation of autolysosomes.^164^, ^165^ Cycloheximide inhibits starvation‐induced autophagy through activation of mTOR signaling.^164^ Previous preclinical studies exposed that cycloheximide promotes the expression of insulin‐like growth factors in hepatoma cell lines.^166^, ^167^ In addition, cycloheximide reduces glucose transporters in adipocytes.^168^ Therefore, cycloheximide has detrimental effect on glucose homeostasis and insulin signaling.

#### 3.2.3 Bafilomycins

Bafilomycins are macrolide antibiotics produced by *Streptomycetes*. Bafilomycins have a wide‐range biological activities including antifungal, antiparasitic, antitumor, and immunosuppressant.^169^ Bafilomycin A1 inhibits autophagy leading to mitochondrial dysfunction and cell death, particularly, bafilomycin A1 block vacuolar type H‐ATPase (V‐ATPase) enzyme, which is responsible for acidification of lysosomes and other intracellular organelles.^170^ It has been shown that V‐ATPase inhibits insulin release, and the use of bafilomycin A1 for 1 week attenuates renal gluconeogenesis and improves glucose homeostasis in rats with T2D.^171^ However, transplacental exposure to bafilomycin A1 inhibits pancreatic organogenesis and accelerates diabetes in mice.^172^ Moreover, the expression of V‐ATPase is downregulated in T2D patients.^173^ Therefore, bafilomycin A1 has a detrimental effect on the T2D outcomes by inhibiting autophagy.

**FIGURE 6:**
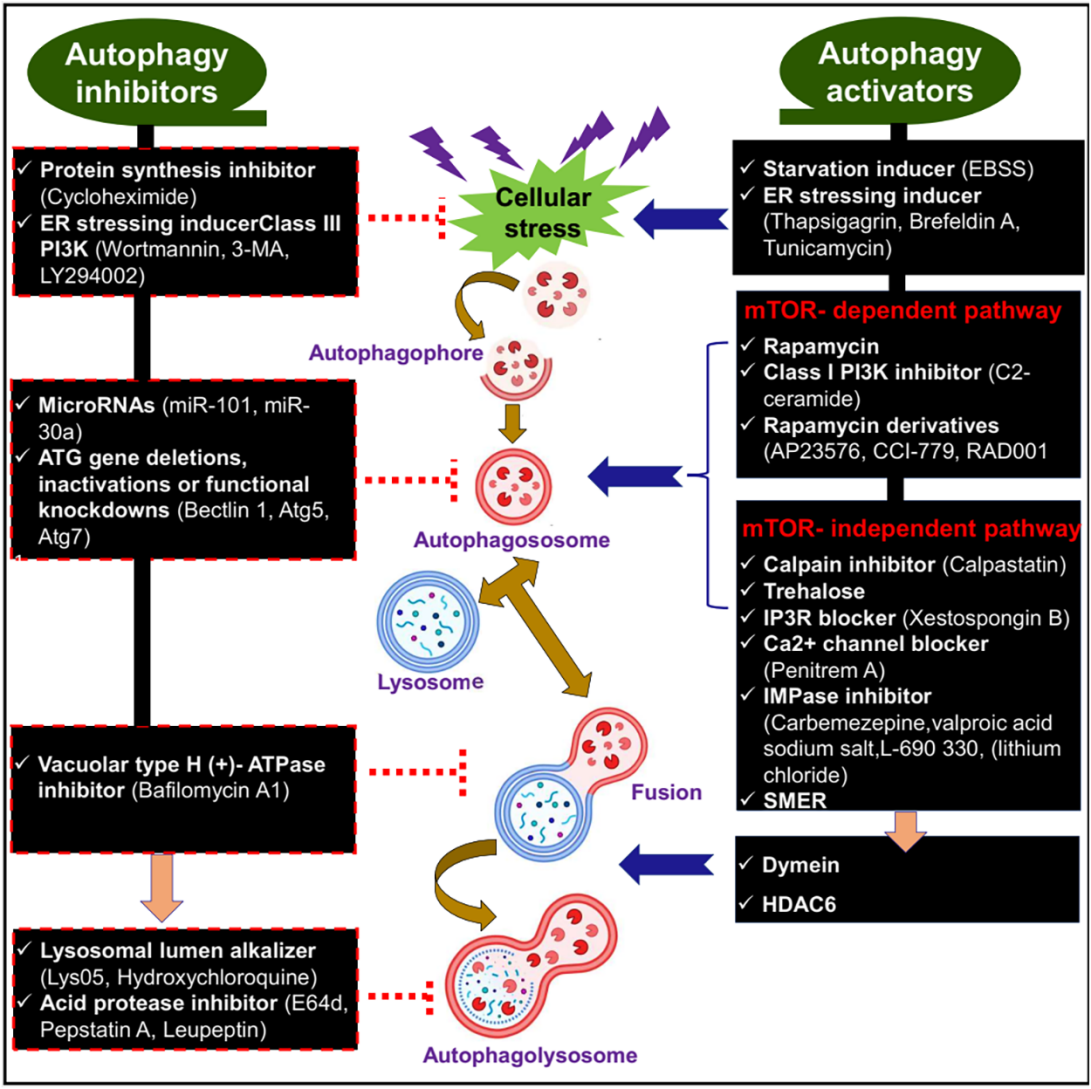
Action of autophagy activators and inhibitors: Autophagy activators inhibit cellular stress and induce autophagosome formation and fusion of autophagosome with the lysosomes; the reverse is done by autophagy inhibitors.

Different sites of actions are affected by autophagy activators and inhibitors (Figure 6).

Taken together, both autophagy activators and inhibitors have conflicting effects regardless of autophagy function.

## 4 CONCLUSIONS AND PERSPECTIVES

T2D is a chronic metabolic disorder associated with dysfunction autophagy pathway Basal autophagy under normal physiological conditions seems to be protective against the development of pancreatic β‐cell dysfunction. During the development of IR and overt T2D with the development of chronic hyperglycemia and associated ER stress, autophagy is activated as a compensatory pathway against oxidative stress and inflammatory to preserve homeostasis of pancreatic β cell. However, in late T2D autophagy is over‐activated due to unresolved oxidative stress leading to the induction of pancreatic β‐cell apoptosis. Therefore, autophagy plays a double‐edged sword role in T2D seeming protective in the early stage and detrimental in the late stage. Therefore, autophagy activators improve defective autophagy in early T2D preventing further deteriorations. However, autophagy inhibitors seem to produce a protective effect in late T2D to prevent exaggerated autophagy which implicated in the induction of pancreatic β‐cell apoptosis. Most autophagy activators seem to have protective effects; however, the majority of autophagy inhibitors have detrimental effects. Collectively, autophagy activators improve pancreatic β‐cells and reduce IR in T2D. In this claim, repurposing of natural products that have potential effects on the autophagy process as adjuvant treatment with antidiabetic agents could be promising as a novel therapeutic strategy. Preclinical and clinical studies are warranted in this concern.

## AUTHOR CONTRIBUTIONS

A.T.Z., H.M.A.‐k., and A.I.A.‐G. collected the related research literature and papers and drafted the manuscript. A.A., M.P., A.A.‐F., Y.H.A.E., M.H.Z., and G.E.‐S.B. participated in the design, editing, and revising the manuscript draft. G.E.‐S.B. contributed to conceptualization. All authors have approved and read the final manuscript.

## FUNDING INFORMATION

Open Access funding enabled and organized by Projekt DEAL. This work was supported by the University of Witten‐Herdecke Germany.

## CONFLICT OF INTEREST STATEMENT

The authors declare no conflicts of interest.
